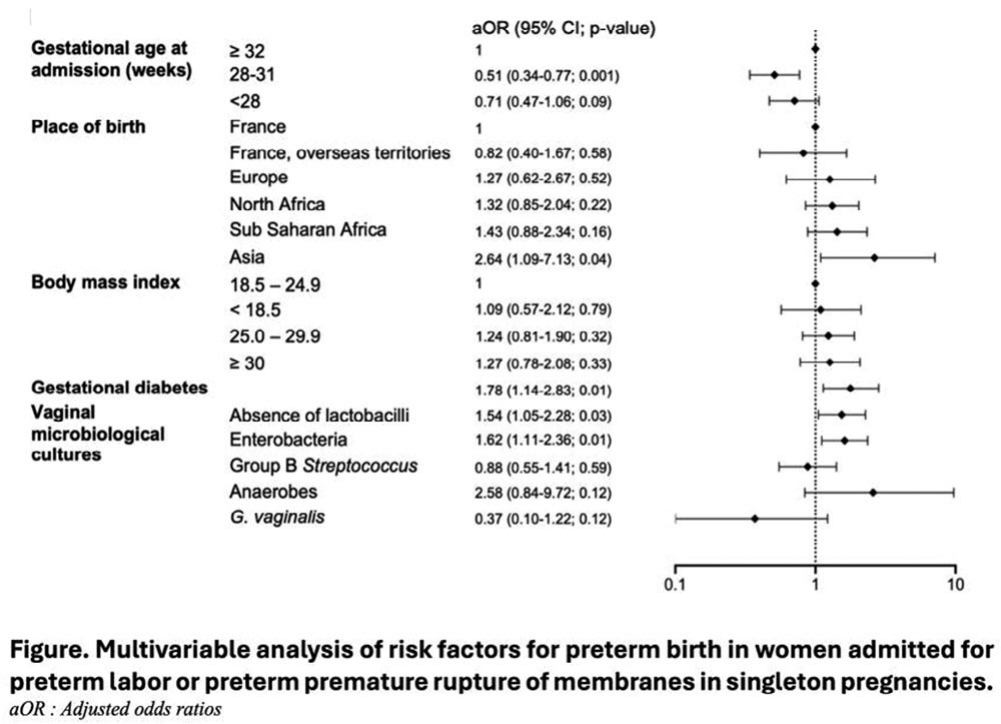# Oral Concurrent Session 6 – Prematurity and Newborn: Friday, January 31, 2025 1:30 PM – 4:00 PM

**DOI:** 10.1002/pmf2.12016

**Published:** 2025-01-05

## 67 | Azithromycin to Prevent Stillbirths and Infant Deaths in Mali: A 2x2 Factorial Placebo‐Controlled Randomized Trial

Karen Kotloff^1^; Amanda J. Driscoll^1^; Fadima Haidara^2^; Lawrence Moulton^3^; Jason Bailey^1^; Ousmane Samake^2^; Tiecoura Bocoum^2^; Jane Juma^2^; Awa Traore^2^; Mamadou Diallo^2^; Collins Okello^2^; Uma Onwuchekwa^2^; Mamoudou Kodio^2^; Yuji Chen^1^; Emily Deichsel^1^; Matthew Finholt‐Daniel^4^; Robert L. Goldenberg^5^; Fleesie Hubbard^1^; Rebecca Maguire^1^; Melissa Page^4^; David Plotner^4^; Milagritos Tapia^1^; Dilruba Nasrin^1^; Samba Sow^2^

*^1^University of Maryland, School of Medicine, Baltimore, MD; ^2^Centre pour le Développement des Vaccins, Mali, Bamako, Bamako; ^3^Johns Hopkins, Bloomberg School of Public Health, Baltimore, MD; ^4^RTI International, Durham, NC; ^5^Columbia University School of Medicine, New York, NY*

1:30 PM ‐ 1:45 PM

**Objective**: To determine if oral azithromycin (AZ) delivered to pregnant women and infants in a high mortality setting reduces stillbirths and infant deaths up to 12 months, compared to placebo.

**Study Design**: We conducted a randomized 2x2 factorial placebo‐controlled trial in 3 districts of Mali, West Africa, to evaluate the efficacy of 2 AZ regimens to prevent stillbirths and/or infant deaths. Pregnant women were recruited at antenatal care (ANC) visits and randomized 1:1:1:1 to one of 4 treatment arms: maternal and infant AZ (AA), maternal AZ and infant placebo (AP), infant placebo and maternal AZ (AP), or maternal and infant placebo (PP). They received up to 3 single 2g doses of AZ or placebo: upon enrolling at ≥14 weeks gestation, at the next routine ANC visit, and at delivery. Their infants received two single 20mg/kg doses of AZ or placebo at routine vaccination visits at approximately 6 and 14 weeks of age. The primary outcome for the maternal intervention was a composite of stillbirth and infant death to up 12 months of age. The primary outcome for the infant intervention was infant death from the time of the first infant dose up to 12 months of age.

**Results**: We enrolled 49,675 pregnant women between Sep 24, 2020, and Feb 27, 2023. The hazard ratio (HR) for stillbirths and infant deaths was 0.94 (95% CI 0.89, 1.00; p‐value = 0.058) in the maternal AZ arms compared to the maternal placebo arms, and 0.90 (0.72, 1.12) for the infant mortality outcome comparing the infant AZ arms to placebo arms. In subgroup analyses, maternal AZ was associated with reduced stillbirth and infant deaths in women with height < 160 cm (0.87; 95% CI 0.79, 0.96) with parity ≥4 (0.86; 95% CI 0.78, 0.95), and for those in Kignan district (0.77; 95% CI 0.65, 0.90). In Kignan, stillbirths were reduced by 19% (risk ratio 0.80; 95%CI 0.65, 0.99) and neonatal deaths 0‐27d by 40% (HR 0.60; 95%CI 0.43, 0.82).

**Conclusion**: Maternal AZ could reduce stillbirths and infant deaths if targeted to high‐risk groups in high mortality settings. The benefit of AZ delivered to infants at routine vaccination visits is uncertain.

## 68 | The Effect of Metformin in Women Who Received Betamethasone on Maternal Hyperglycemia and Neonatal Hypoglycemia

Enav Yefet^1^; Manal Massalha^2^; Gil Talmon^1^; Aminet Labay^1^; Marian matanis^1^; Erez Sleman^1^; Rima nassra^1^; Maya Frank Wolf^3^; Inshirah Sgayer^4^; Lior Lowenstein^4^; Zohar Nachum^2^

*^1^Tzafon Medical Center, Poriya, HaZafon; ^2^Emak Medical Center, Afula, HaZafon; ^3^Galilee Medical Center, Naharyia, HaZafon; ^4^Galilee Medical center, Nahariya, HaZafon*

1:45 PM ‐ 2:00 PM

**Objective**: While betamethasone reduces complications of prematurity, it can cause maternal hyperglycemia and neonatal hypoglycemia. Metformin effectively treats maternal hyperglycemia and has been shown to decrease neonatal hypoglycemia in women with gestational diabetes mellitus.

This study examines metformin's impact on maternal hyperglycemia and neonatal hypoglycemia in preterm deliveries in women administered betamethasone.

**Study Design**: This multicenter, open‐label, randomized controlled trial was conducted from 2020 to 2024 at three medical centers. Women with diabetes were excluded. Pregnant women receiving betamethasone between 24 and 36.5 weeks of gestation due to increased preterm delivery risk were randomly assigned to either metformin (425 mg three times daily before meals and 850‐1700 mg at 10 p.m.) or no treatment. The treatment lasted up to 48 hours following the first betamethasone dose. Capillary glucose was measured before meals (pre‐prandial), 90 minutes after starting meals (post‐prandial), and at 10 p.m.

Primary outcomes were the mean maternal daily glucose values and neonatal hypoglycemia in preterm deliveries. To show a reduction in neonatal hypoglycemia from 40% in the control group to 15% in the metformin group, 98 neonates were needed (two‐tailed alpha = 0.05, power of 80%). To demonstrate a mean difference of 5 mg% with a 10 mg% standard deviation in daily glucose levels, 156 women were required (two‐tailed alpha = 0.025, power of 80%).

**Results**: A total of 84 and 85 women in the metformin and control groups, respectively, were analyzed. Preterm neonates included 48 from the metformin group and 58 from the control group.

In the metformin group, mean maternal daily and postprandial glucose values were significantly lower than in the control group. The rate of neonatal hypoglycemia was lower in the metformin group compared to the control group (10 (21%) vs. 23 (40%), P = 0.037, relative risk 0.53, 95% CI 0.28‐0.99). Mild adverse effects were reported by 14% of women.

**Conclusion**: Metformin is safe and effective in preventing steroid‐induced maternal hyperglycemia and neonatal hypoglycemia.

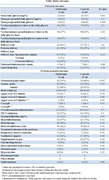

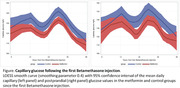

## 69 | Fully Quantitative Cervical Remodeling: Race Group Differences Responsibilities and Cautions

LeAnn A. Louis^1^; Sarah M. Dwyer^1^; Methodius G. Tuuli^2^; Adam K. Lewkowitz^2^; Julie Tumbarello^1^; Emily Dively, BSN^3^; Madeline Felske^1^; Wendy Sparks^1^; Anita M. Malone^1^; Peinan Zhao^4^; Molly J. Stout^5^

*^1^University of Michigan Hospital, Ann Arbor, MI; ^2^Women & Infants Hospital of Rhode Island and Alpert Medical School of Brown University, Providence, RI; ^3^Washington University School of Medicine, St. Louis, MO; ^4^Washington University, St. Louis, MO; ^5^University of Michigan Medical Center, Ann Arbor, MI*

2:00 PM ‐ 2:15 PM

**Objective**: We aimed to examine fully quantitative cervical tissue stiffness patterns in pregnancy by race groups across 3 academic centers.

**Study Design**: We invented fully quantitative cervical elastography (FQ‐CES), which yields a numeric value of cervical tissue stiffness (Young's modulus) and allows comparison within and across patients over time.

This is a prospective longitudinal cohort of singleton pregnancies at 3 academic centers. We measured FQ‐CES in all trimesters in asymptomatic individuals. We defined starting cervical stiffness and softening rate over pregnancy by term and preterm birth (PTB) in the total cohort and across race groups using mixed regression models. Race was self‐reported.

**Results**: 380 individuals were included. Those with PTB start pregnancy with softer cervixes (29.2% lower Young's modulus, p = 0.03). Among Black individuals, the cervix started firmer (33.5% higher Young's modulus in the first trimester, p = 0.03) and had a faster softening rate (8.0% vs 6.5% per week, p = 0.002) compared to non‐Black individuals. Within those with PTB, Black individuals start pregnancy with firmer cervixes compared to non‐Black individuals (240% higher in Young's modulus, p = 0.01) meaning that even with a firmer cervix Black individuals still had increased risk for PTB.

**Conclusion**: PTB differentially affects minoritized populations. While those with PTB generally start pregnancy with softer cervixes, racial differences exist, with Black individuals starting firmer and softening faster. This may be due to racial differences in environmental factors and their impact on the pathway to PTB. If normative values were defined from the total cohort, estimates could underestimate risk in Black individuals (starting pregnancy firmer, placing them in a lower risk category). While differential interpretation of screening and diagnostic tests has perpetuated racism and risk in minoritized groups, we caution that overlooking differences mediated by racism and related factors may have unintended consequences of underestimating risk in at risk groups.

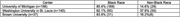

## 70 | Neonatal Outcomes After Caesarean Versus Vaginal Birth: Population‐based Cohort Study of Extremely Preterm Breech Singletons

Yanchen Wang^1^; Pasqualina Santaguida^1^; Sameer Parpia^1^; Fabiana Bacchini^2^; Prakeshkumar S. Shah^3^; Kellie Murphy^3^; K. S. Joseph^4^; Sandesh Shivananda^5^; Sarah D. McDonald^1^

*^1^McMaster University, Hamilton, ON; ^2^Canadian Premature Babies Foundation, Toronto, ON; ^3^University of Toronto, Toronto, ON; ^4^School of Population and Public Health, University of British Columbia, Vancouver, BC; ^5^University of British Columbia, Vancouver, BC*

2:15 PM ‐ 2:30 PM

**Objective**: To determine the association of Caesarean (versus vaginal) birth with neonatal outcomes among extremely preterm breech singletons admitted into a neonatal intensive care unit.

**Study Design**: This retrospective population‐based cohort study included all breech singletons between 23 and 27 weeks’ gestation, who received active resuscitation and were admitted to one of 30 level‐III neonatal intensive care units within the Canadian Neonatal Network from 2010 to 2022. We excluded outborn singletons and those with major congenital anomalies. The primary outcome was a composite of neonatal death, birth trauma, or severe neurological complications. The primary analysis to compare those born by Caesarean section versus vaginally was carried out using a generalized estimating equation based Poisson regression model with propensity score and instrumental variable analyses as sensitivity analyses.

**Results**: Of the 3332 extremely preterm breech singletons included in the study population, 2778 (83.4%) were born by Caesarean section. The adjusted incidence of the neonatal composite outcome was 26% following Caesarean section, versus 34% following vaginal birth (adjusted relative risk [aRR]: 0.77, 95% confidence interval [CI]: 0.63, 0.95). The reduction in the neonatal outcome following Caesarean section persisted after propensity score matching (aRR: 0.69, 95% CI: 0.53, 0.89) and instrumental variable analysis (aRR: 0.81, 95% CI: 0.67, 0.98). In the subgroup of singletons receiving optimized perinatal care (including antenatal corticosteroids, magnesium sulfate, and deferred cord clamping), the adjusted reduction in the neonatal composite outcome was 0.63 (95% CI: 0.41, 0.98), and in those without optimized perinatal care (aRR: 0.80, 95% CI: 0.63, 1.01).

**Conclusion**: Most (83%) extremely preterm breech singletons were born by Caesarean section with an associated reduction in mortality/serious morbidity. This association was robust and consistently observed in analyses addressing potential confounding through different methods, and in those receiving optimized care or not.

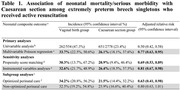

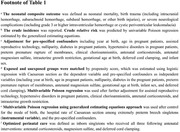

## 71 | The Role of Cervical Elastography for the Prediction of Spontaneous Preterm Birth

Anne‐Sophie Lafortune^1^; Louise Ghesquiere^2^; Paul Guerby^3^; Marie‐Laurence Côté^1^; Genevieve Marcoux^4^; Annie Beaudoin^4^; Emmanuel Bujold^1^

*^1^Université Laval, PQ; ^2^Université de Lille, Lille, Nord‐Pas‐de‐Calais; ^3^Université de Toulouse, Midi‐Pyrenees; ^4^CHU de Québec, CHU de Québec, PQ*

2:30 PM ‐ 2:45 PM

**Objective**: Cervical elastography is a tool for the prediction of spontaneous preterm birth (sPTB). We aimed to estimate its role in addition to cervical length (CL) in low‐risk pregnancies.

**Study Design**: We performed a prospective study including nulliparous participants who underwent transvaginal ultrasound at 20‐23 weeks. Among those with CL >25 mm, stiffness of the cervix using strain elastography was evaluated using the following parameters: internal os strain (IOS), external os strain (EOS), and the hardness ratio (HR). Operators and patients remained blinded to the results. Receiver operator characteristics (ROC) curves along with non‐parametric statistical analyses were used to estimate the predictive values of each parameter.

**Results**: Among 991 eligible participants with CL >35mm, 32 (3.2%) had sPTB including 9 (0.9%) with sPTB before 35 weeks. Among all parameters, we observed that EOS was highly associated with sPTB< 35 weeks (p< 0.001; figure) with an optimal cut‐off of 0.39, along with a HR< 40% (p = 0.01) and CL< 35 mm (p = 0.02). The combination of a CL< 35mm and EOS >0.39 was associated with a greater risk of sPTB (RR: 5.5; 95%CI: 1.6–18.5) and mainly sPTB< 35 weeks (RR: 12.6; 95%CI: 1.4–112; p< 0.01).

**Conclusion**: Mid‐trimester cervical elastography (EOS and HR) is highly associated with the risk of sPTB and particularly sPTB< 35 weeks when CL is >25 mm. Its use in addition to CL should be rapidly considered in clinical practice.

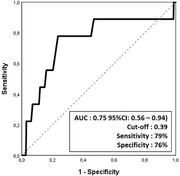

## 72 | Corticosteroids and Neonatal Hypoglycemia among Pregnant Individuals with Diabetes: Effect of Gestational Age at Delivery

Ruby Lin; Cande V. Ananth; Todd J. Rosen; On behalf of the MOMPOD Consortium

*Rutgers Robert Wood Johnson Medical School, New Brunswick, NJ*

2:45 PM ‐ 3:00 PM

**Objective**: While antenatal steroids have been linked to neonatal hypoglycemia (NNH) among pregnant patients with diabetes, the impact of gestational age is unclear. We hypothesize that ACS for fetal lung maturity for patients with pregestational and early gestational diabetes is associated with a higher risk of NNH, but early preterm delivery < 34 weeks mediates this risk.

**Study Design**: This is a secondary analysis of MOMPOD, a multicenter, a randomized control trial of 831 pregnant patients with preexisting T2DM or diabetes diagnosed before 23 weeks gestation treated with insulin alone or insulin and metformin. All participants exposed to ACS were compared to those who were not. The primary outcome was NNH defined as blood glucose < 40 mg/dl or need for IV dextrose treatment. We undertook a causal mediation analysis to disentangle the effects of steroids on neonatal hypoglycemia that operate through early preterm delivery (< 34 weeks) versus the effect that is independent of early preterm delivery. We estimated the confounder‐adjusted relative risk (RR) with a 95% confidence interval (CI) through log‐binomial regression models.

**Results**: Of the 765 patients with available data, 115 received ACS. Two‐thirds (68%, n = 78) of those who received ACS had NNH compared to 42% (n = 273) of those who did not receive ACS (Table 1). ACS exposure was associated with a 1.82‐fold (95% CI 1.40‐2.51) increased risk of  NNH. 82% of this increased risk of NNH associated with corticosteroid exposure was mediated through delivery at < 34 weeks (Table 2).

**Conclusion**: ACS exposure is associated with a 1.8‐fold adjusted risk of NNH, but the majority of this effect is mediated through preterm delivery < 34 weeks. Efforts to design secondary clinical interventions and protocols to reduce the burden of NNH associated with ACS exposure among neonates < 34 weeks born to pregnant patients with diabetes would be of benefit.

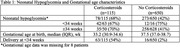

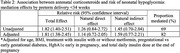

## 73 | Pro‐inflammatory Vaginal Cytokines, Early PTB, and Infectious Morbidity in Patients with Asymptomatic Cervical Shortening

Tracy A. Manuck; On behalf of the Eunice Kennedy Shriver National Institute of Child Health and Human Development Maternal‐Fetal Medicine Units (MFMU) Network

*University of North Carolina, Chapel Hill, NC*

3:00 PM ‐ 3:15 PM

**Objective**: There is a critical need to improve risk‐stratification of individuals with an asymptomatic short cervical length (CL).

**Study Design**: Secondary analysis of a multicenter, unmasked RCT of pessary vs. usual care, 2017‐2021. Participants with singleton pregnancies, an asymptomatic CL ≤20mm (16+0‐23+6 wks), no prior PTB, and a vaginal swab at enrollment were included. 12 cytokines were quantified using a Luminex assay. The primary outcome was PTB < 35 wks. Secondary outcomes were PTB < 32, < 28, and < 24 wks and maternal (chorio) and neonatal (sepsis ± pneumonia) infectious morbidity. Each cytokine was classified as ‘high’ or ‘low’ using a numeric cut point that optimized the sensitivity and specificity for PTB < 35 wks. A pro‐inflammatory cytokine score was calculated for each patient by adding +1 point per ***high* pro‐**inflammatory (IL‐1𝛂, IL‐1β, IL‐2, IL‐6, IL‐8, GM‐CSF, TNF‐𝛂, VEGF) and +1 point per ***low *anti‐**inflammatory (IL‐1RA, IL‐4, IL‐10, G‐CSF) cytokine. Data were analyzed by logistic regression; AUC analyses evaluated outcome prediction of clinical‐only, cytokine‐only, and clinical+cytokine models.

**Results**: 531 of 544 RCT participants met inclusion criteria; 37% had PTB < 35 wks; 7% had maternal and 22% had neonatal infectious morbidity. Cytokines were collected at a median 21.7 (IQR 20.7, 23.0) wks. Cytokine scores ranged 1‐9 [median 4, IQR (3,5)] and were higher for those with PTB < 35 wks vs. >35 wks (4.7 vs. 4.0, p< 0.001). In regressions, the composite cytokine score was associated with PTB < 35, < 32, < 28, and < 24 wks and maternal and neonatal infectious morbidity. Outcome prediction in clinical+cytokine models did not statistically improve vs. clinical‐only models (Table). In Kaplan‐Meier analysis, higher cytokine scores were associated with earlier delivery (Figure; log‐rank *p*< 0.001).

**Conclusion**: A mid‐trimester pro‐inflammatory vaginal cytokine milieu is associated with PTB and infectious morbidity in patients with asymptomatic short CL. Non‐invasive vaginal cytokine quantification may be an additional way to identify which individuals have pathologic vs. incidental CL shortening.

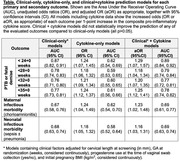

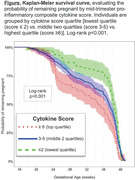

## 74 | Comparison of Polyester Fiber versus Polypropylene Suture Materials in Physical‐Exam Indicated Transvaginal Cerclages

Daniel J. Martingano^1^; Amanda F. Francis Oladipo^2^; Marwah Al‐Dulaimi^3^; Sandra Kumwong^4^; Andrea Ouyang^5^; Lauren Cue^6^; Ashley Nguyen^3^; Shailini Singh^7^; Alexander Ulfers^8^; Mark Rebolos^3^; Kristin Cohen^9^; Donald Morrish^3^; Iffath A. Hoskins^10^; Francis X. Martingano^11^

*^1^St. John's Episcopal Hospital‐South Shore and William Carey University College of Osteopathic Medicine, Far Rockaway, NY; ^2^Hackensack University Medical Center, Hackensack, NJ; ^3^St. John's Episcopal Hospital‐South Shore, Far Rockaway, NY; ^4^Touro College of Osteopathic Medicine‐Harlem Campus, New York, NY; ^5^William Carey University, Hattiesburg, MS; ^6^Rutgers University and the Jersey City Medical Center, Jersey City, NJ; ^7^AtlantiCare Regional Medical Center, Pamona, NJ; ^8^Walter Reed National Military Medical Center, Bethesda, MD; ^9^RWJBarnabas Health ‐ Trinitas Regional Medical Center, Elizabeth, NJ; ^10^Albert Einstein College of Medicine ‐ Montefiore Medical Center, New York, NY; ^11^NYU Grossman School of Medicine ‐ NYU Brooklyn, New York, NY*

3:15 PM ‐ 3:30 PM

**Objective**: Polyester fiber (Mersilene) and polypropylene (Prolene) suture materials are the most common type used when performing transvaginal physical‐exam indicated cerclages, yet there is insufficient evidence regarding their comparative effectiveness in prolonging gestational latency. This study sought to determine and compare this effectiveness in pregnancies requiring a physical‐exam indicated transvaginal cerclage using either Mersilene or Prolene suture material.

**Study Design**: We conducted a multi‐center, prospective observational study from 7/2022 to 7/2024 comparing all pregnancies complicated by cervical insufficiency requiring physical‐exam indicated transvaginal cerclage at a gestational age range of 160/7 to 236/7 weeks. Patients with multiple gestations, prior vaginal progesterone, and concurrent antibiotic administration apart from perioperative use were excluded. All patients received perioperative cefazolin and postoperative indomethacin. The primary outcomes included completed weeks gestation at time of delivery, early‐preterm (306/7–336/7 weeks), late‐preterm (340/7–366/7 weeks), early‐term (370/7–386/7 weeks), and full term deliveries (390/7–406/7), as respective, discrete events.

**Results**: The study included 302 patients with 143 receiving Mersilene and 159 receiving Prolene. Demographic factors were not significantly different. Kaplan‐Meier survival analysis noted increased gestational latency in patients receiving Prolene (p = 0.002, Figure 1). Patients receiving Mersilene had higher rates of early‐preterm deliveries (46.4% v. 29.3%, p = 0.001) with a 21% increased risk in confounder adjusted models (RR = 1.21, 95% CI 1.01‐1.39, p = 0.021) and lower rates of full‐term deliveries (10.5% v. 31.1%, p = 0.003) with a 11% decreased likelihood in confound adjusted models (RR = 0.89, 95%CI 0.81‐0.98, p = 0.004). Rates of late‐preterm and early‐term deliveries were not significantly different.

**Conclusion**: Prolene suture may be a more beneficial choice of suture material for improving gestational latency with physical‐exam indicated transvaginal cerclages.

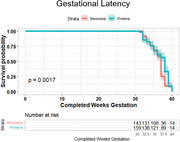

## 75 | Antimuscarinic Receptor Blockade Reduces Uterine Muscle Contractions Potentially Providing A Novel Treatment For Preterm Labor

Anthony G. Visco^1^; Zachary Visco^2^; Chad Grotegut^3^; Cristina Linde^4^; Timothy Westfall^4^; Friederike Jayes^5^

*^1^NinoMed, LLC, Chapel Hill, NC; ^2^University of North Carolina, Chapel Hill, NC; ^3^Wake Forest University, Wake Forest, NC; ^4^Reprocell, Beltsville, MD; ^5^Duke University, Durham, NC*

3:30 PM ‐ 3:45 PM

**Objective**: The bladder and the uterus have striking physiologic and anatomic similarities. Anticholinergic medications are commonly used to treat “overactive bladder” by targeting muscarinic receptors. The uterus also contains M2 and M3 muscarinic receptors; however, current management of preterm labor does not target these receptors. We assessed whether muscarinic blockade with oxybutynin reduces both spontaneous and oxytocin‐induced uterine muscle contractions.

**Study Design**: Longitudinal strips of uterine muscle from pregnant rats (17‐19 days gestation) were mounted on isometric test apparatuses to directly measure uterine muscle contractions. The effect of oxybutynin on both spontaneous and on oxytocin‐induced (1nM) uterine muscle contractility was tested.  We measured both the area under the curve (integral) and frequency of the uterine muscle contractions with cumulatively higher concentrations of oxybutynin (100nM, 300nM, 1uM, 3uM, 10uM, 30uM, 100uM) compared to water vehicle control. Measurements are presented as % of baseline. Two‐Way ANOVA with Dunnett's post hoc test was performed.

**Results**: Uterine muscle strips harvested from pregnant rats consistently showed spontaneous muscle contractions. Oxybutynin, at concentrations ≥10µM, resulted in a statistically significant decrease in both spontaneous uterine muscle strength (integral) and contraction frequency compared to vehicle controls. (See Figures 1 and 2).

Oxytocin‐induced uterine muscle contractions were also affected by oxybutynin. At concentrations ≥10µM, uterine muscle strength (integral) was reduced (p< 0.01), but no difference was observed in contraction frequency compared to controls. (Data not shown).

**Conclusion**: Oxybutynin significantly reduces spontaneous uterine muscle strength (integral) and contraction frequency. It reduces oxytocin‐induced uterine muscle strength (integral) but not contraction frequency. Antimuscarinic blockers warrant future clinical investigation and may represent an exciting new paradigm in the treatment of preterm labor and “overactive uterus”.

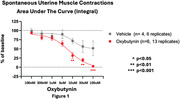

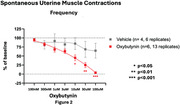

## 76 | Vaginal Microbiota as a Predictor of Preterm Birth: a Prospective Cohort Study

Laura Lesimple^1^; Jessica Rousseau Rousseau^1^; Luce Landraud^1^; Céline Plainvert^1^; Nathalie Grall^1^; Francois Goffinet^2^; Pierre‐Yves Ancel^1^; Christophe Pannetier^3^; Laurent Mandelbrot^4^; Asmaa Tazi^1^

*^1^Assistance Publique Hôpitaux de Paris, Paris, Ile‐de‐France; ^2^Université Paris Cité, Inserm, Centre for Research in Epidemiology and StatisticS (CRESS), Obstetrical Perinatal and Pediatric Epidemiology Research Team (EPOPé), Paris, Ile‐de‐France; ^3^Assistance Publique Hôpitaux de Paris, Paris, Rhone‐Alpes; ^4^Hôpital Louis Mourier, APHP, Université Paris Cité, Colombes, Ile‐de‐France*

3:45 PM ‐ 4:00 PM

**Objective**: To identify microbiota signatures of preterm labor and premature rupture of membranes (PPROM).

**Study Design**: A prospective cohort study in Paris, France. Four groups were enrolled, a control group and 3 groups at risk of intrauterine infection or preterm birth: prelabor rupture of membranes at term, preterm labor, and PPROM. Vaginal swabs were collected at admission and were analyzed using culturomics. The main outcome was preterm birth.

**Results**: 2476 women were enrolled, including 1068 controls, 477 with prelabor rupture of membranes at term, 495 with preterm labor, and 436 with PPROM. Several vaginal microbiota signatures were identified as correlated with pregnancy outcome. In multivariable analysis adjusted for maternal and obstetrical risk factors, lactobacilli depletion and enterobacteria were risk factors for preterm birth, especially in singleton pregnancies (aOR 1.54, 95% CI 1.05‐2.28 and aOR 1.62, 95% CI 1.11‐2.36, respectively, figure). Additionally, preterm labor was associated with lactobacilli depletion (aOR 1.49, 95% CI 1.08‐2.06), enterobacteria (aOR 1.86, 95% CI 1.36‐2.53) and G. vaginalis (aOR 4.62, 95% CI 1.86‐13.34); PPROM with lactobacilli depletion (aOR 2.04, 95% CI 1.49‐2.80) and enterobacteria (aOR 2.38, 95% CI 1.74‐3.24). Prelabor rupture of membranes at term was associated with enterobacteria (aOR 1.97, 95% CI 1.46‐2.65) and Gardnerella vaginalis (aOR 5.19, 95% CI 2.22‐13.78).

**Conclusion**: In this large cohort, *G. vaginalis* was associated with preterm labor and prelabor rupture of membranes at term, while lactobacilli depletion and enterobacteria were risk factors for PPROM and preterm birth. The supports the need for further studies of adapted antibiotics and probiotics to restore a healthy microbiota for preventing preterm birth.